# Biomechanical characteristics of proficient free-throw shooters—markerless motion capture analysis

**DOI:** 10.3389/fspor.2023.1208915

**Published:** 2023-08-02

**Authors:** Dimitrije Cabarkapa, Damjana V. Cabarkapa, Jonathan D. Miller, Tylan T. Templin, Lance L. Frazer, Daniel P. Nicolella, Andrew C. Fry

**Affiliations:** ^1^ Jayhawk Athletic Performance Laboratory—Wu Tsai Human Performance Alliance, Department of Health, Sport and Exercise Sciences, University of Kansas, Lawrence, KS, United States; ^2^ Higuchi Biosciences Center, University of Kansas, Lawrence, KS, United States; ^3^ Southwest Research Institute, San Antonio, TX, United States

**Keywords:** sport, performance, kinematic, kinetic, assessment, shooting

## Abstract

The winning game outcome in basketball is partially contingent on the team's ability to secure and make more free-throw shooting attempts, especially close to the end of the game. Thus, the purpose of the present study was to perform a comprehensive biomechanical analysis of the free-throw shooting motion to examine differences between (a) proficient (≥70%) and non-proficient shooters (<70%) and (b) made and missed free-throw shoots within the proficient group of shooters. Thirty-four recreationally active males with previous basketball playing experience attempted ten consecutive free-throw shots (4.57 m), with a 10–15 s rest interval between each shot. An innovative three-dimensional markerless motion capture system (SwRI Enable, San Antonio, TX, USA) composed of nine high-definition cameras recording at 120 Hz was used to capture and analyze the biomechanical parameters of interest. Independent *t*-tests and Mann–Whitney *U* tests were used to examine a presence of statistically significant differences. The findings of the present study reveal that proficient free-throw shooters performed the shooting motion in a more controlled manner by having significantly lower knee and center of mass peak and mean angular velocities. Also, proficient shooters attained a significantly greater release height and had less forward trunk lean when compared to non-proficient shooters at the time point of the ball release. Moreover, despite being beneficial for improvements in shooting accuracy, our findings suggest that overemphasizing the release height may be in certain instances counterproductive, as it may lead to more missed than made free-throw shots within the proficient group of shooters.

## 1. Introduction

Shooting efficiency is one of the key performance parameters capable of differentiating winning from losing game outcomes on various levels of basketball competition (1–7). A recently published study found that field-goal, three-point, and free-throw shooting percentages were all significantly greater in winning than losing teams during both regular and post-season competitive periods (3). However, unlike other types of shooting motions (e.g., lay-up, jump shot), the free-throw shot is an uncontested scoring opportunity that allows players to score points without the presence of a defender. It has been found that the desired game outcome is partially contingent on the team's ability to secure and make more free-throw shooting attempts, especially close to the end of the game (8, 9). Thus, it is understandable why sports scientists and coaches place a considerable emphasis on studying and developing basketball players' free-throw shooting efficiency.

As a complex motor skill that requires coordination of upper and lower-body extremities, a broad spectrum of biomechanical parameters of free-throw shooting motion have been examined in the scientific literature, using both theoretical and applied research design models (10–19). For example, Brancazio (15) has indicated that the ball release height is of critical importance for the success of the free-throw shooting motion. Although the ball is generally released ∼30–60 cm above the shooter's head, additional increases in release height would allow for a larger margin of error by reducing launching speed and force requirements (15). Offering further support to previously mentioned findings, Tran & Silverberg (19) found that the estimated optimal release angle is ∼52 degrees. Also, it should be noted that applying an appropriate release angle can have a notable impact on the ball trajectory. It is suggested that the best free-throw shot does not pass directly through the center of the basket, but rather between the center and the back of the rim (16). In addition, previous research has found that the synergy of elbow and wrist movements close to the time point of ball release is important for the success of the shooting motion (11, 18). Interestingly, experienced players demonstrated an increase in elbow-wrist coordination-variability prior to ball release during missed free-throw attempts (18). This likely occurred after acknowledging that the shooting technique was incorrect as a result of the attempt to make adequate adjustments (18).

Based on the previously mentioned research reports, it is obvious that the majority of scientific literature has been focused on analyzing biomechanical parameters during the release phase of the free-throw shooting motion. Without questioning their impact on optimal shooting performance, the preparatory phase of the shooting motion remains underexamined. Solely being focused on analyzing kinetic and kinematic characteristics at the time point of the ball release may restrict a deeper understanding of all other factors that ultimately lead to an appropriate ball release height and/or release angle. While limited in quantity, several recently conducted studies have focused on addressing this issue (10, 12–14). Cabarkapa et al. (12) have found that lower elbow positioning during the preparatory phase of the shooting motion, resulting from greater flexion in hip, knee, and ankle joints, was a characteristic of proficient free-throw shooters. The same group of authors found that less lateral elbow deviation was also associated with superior free-throw shooting accuracy in college-age male basketball players (13). In addition, Ammar et al. (10) indicated that greater values of the total movement in the knee joint (i.e., preparatory to release phase) were positively associated with a number of made free-throw shots during a learning process.

It should be noted that the majority of the applied research studies used a high-definition video analysis technique or marker and/or sensor-based motion capture systems to examine various biomechanical parameters of interest in free-throw shooting motion (10, 12–14). While being an affordable and practical testing modality, video analysis allows for the assessment of shooting technique solely in a single plane of motion (e.g., sagittal plane). Conversely, marker-based systems are prone to skin movement artifacts and may alter or restrict an individual's normal range of motion (20, 21). Further, the marker characteristics (e.g., size, reflection capacity) and environmental conditions (e.g., lighting) may be additional limiting factors that need to be considered when using marker-based motion capture systems (21, 22). Therefore, with rapid technological development over the last decade, the solution for objective sport-specific performance assessment of free-throw shooting motion may be in markerless motion capture systems that allow unrestricted movement in all planes of motion. This technology has been previously shown as an adequate tool for the assessment of kinetic characteristics of basketball-specific motions (e.g., dunking) and functional movement health screening scores (21, 22–25).

Therefore, to bridge a gap in the scientific literature, the purpose of the present study was to perform a comprehensive biomechanical analysis during both the preparatory and release phases of the free-throw shooting motion by utilizing an innovative markerless motion capture technology to examine differences between (a) proficient and non-proficient free-throw shooters and (b) made and missed shoots within the proficient group of shooters.

## 2. Materials and methods

### 2.1. Participants

Thirty-four recreationally active males (age = 23.9 ± 7.4 years; height = 182.7 ± 7.4; body mass = 80.1 ± 11.6 kg) with >4 years of previous basketball playing experience (e.g., high school, collegiate; 6.5 ± 2.2 years) volunteered to participate in the present study. All participants were free of musculoskeletal injuries that could impair the full joint range of motion and alter the shooting technique. The testing procedures performed in this investigation were previously approved by the University's Institutional Review Board, and all participants signed an informed consent document.

### 2.2. Procedures

Upon arrival at the basketball gym, each participant completed a warm-up procedure consisting of dynamic stretching exercises (e.g., high knees, A-skips, walking lunges, quad pulls, butt kicks) and 10–15 practice shots from self-selected distances. Then, while standing on a standardized basketball free-throw line (4.57 m), each participant attempted ten shots. The basket height (305 cm) and the ball size (74.93 cm) corresponded to National Collegiate Athletic Association (NCAA) regulations standards. To eliminate the possible influence of fatigue, each shot was separated by a 10–15 s rest interval and research assistants were present throughout all testing procedures to complete the rebounding and passing tasks. Also, to minimize any kind of possible distraction, the participants individually completed all testing procedures.

**Figure 1:**
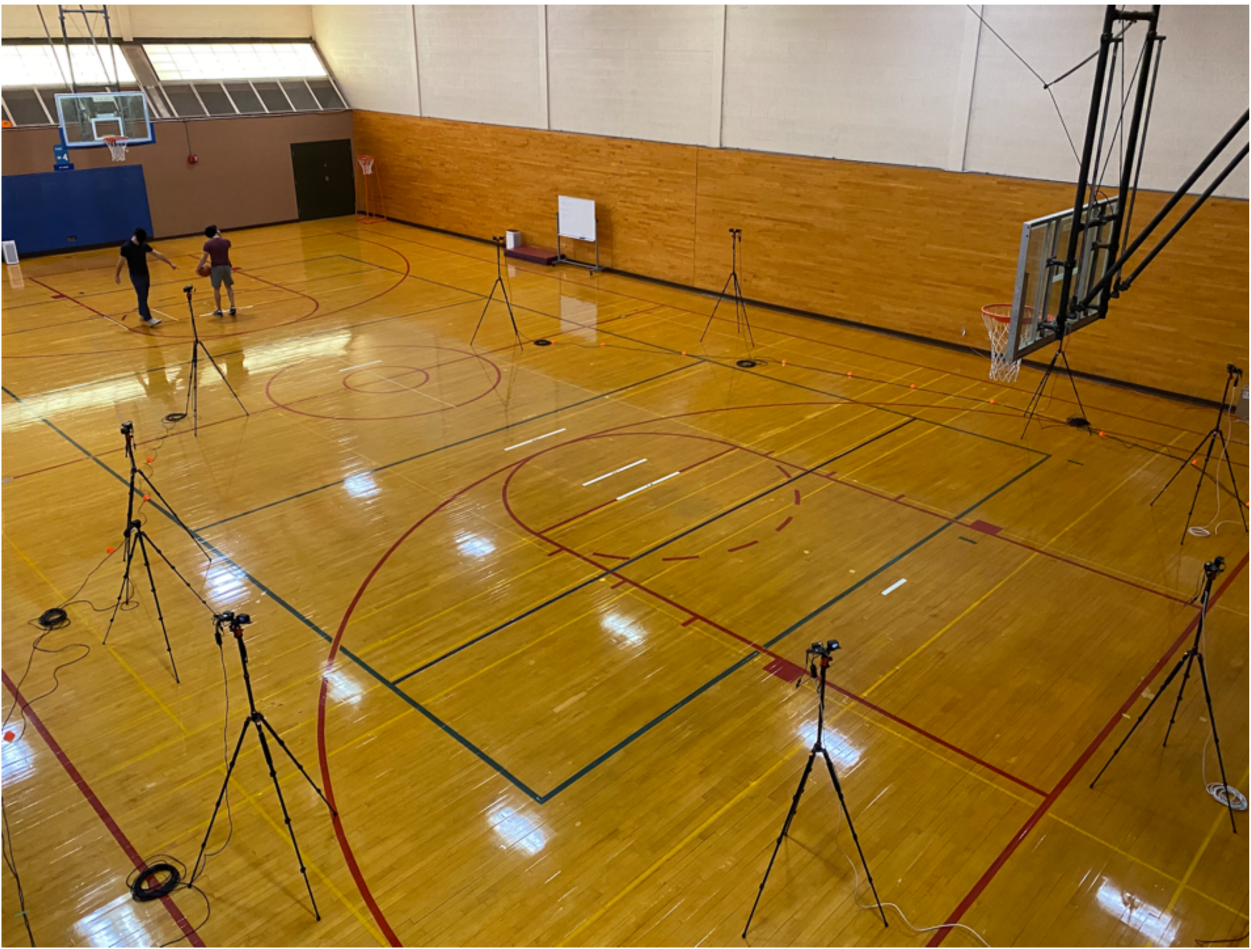
Experimental set-up of the markerless motion capture system.

An innovative three-dimensional markerless motion capture system (SwRI Enable, San Antonio, TX, USA) composed of nine high-definition cameras (Sony Corporation, RXO-II, Tokyo, Japan) was used to capture and analyze the biomechanical parameters of interest. The cameras were positioned to evenly cover the half-court area and allow participants unrestricted movement. Data were collected at 120 Hz and the system was calibrated prior to the start of the data collection. The video recordings were trimmed to start with the preparatory phase and end with the release phase of the free-throw shooting motion. The graphical representation of the experimental set-up is presented in Figure 1.

### 2.3. Variables

The biomechanical parameters examined in the present study were based on previous research reports (10, 12–14, 26–28). The preparatory phase was defined as an initial concentric movement during the shooting motion and the release phase was defined as the time point at which the ball left the shooter's hand. The range of motion (ROM) represents the amount of movement that occurred in each joint between the preparatory to the release phases of the shooting motion (e.g., knee joint ROM, hip joint ROM). The fully extended limb (e.g., knee angle, hip angle, elbow angle) with a value of 180 deg represented a full joint extension. In addition, the mean velocity represents the rate of change in angular displacement (e.g., elbow mean angular velocity) and the peak velocity represents the maximal angular velocity observed in the specific joint between the preparatory and the release phases of the shooting motion (e.g., ankle peak angular velocity). A detailed description of each dependent variable is presented in Table 1.

**Table 1:** Definitions of biomechanical parameters examined in the present study.

Variable	Definition
Release angle	Vertical angle in relation to the ground at which the ball leaves the hand at the release phase of the shooting motion.
Knee angle	Internal angle between the thigh and shank.
Hip angle	Internal angle between the torso and the thigh.
Ankle angle	Internal angle between the shank and the foot.
Elbow angle	Internal angle between the upper arm and forearm.
Center of mass height	Perpendicular distance between the point within the body at which the overall shooter’s mass is located and the ground divided by the participant’s height.
Center of mass velocity	Velocity of the point in the body at which the overall shooter’s mass is located between the preparatory and the release phases of the shooting motion.
Stance width	Distance between the placement of the right and left foot during the preparatory phase of the shooting motion.
Stance alignment	Vertical feet alignment during the preparatory phase of the shooting motion. A positive value indicates that the right foot is placed more forward and a negative value that the left foot is placed more forward.
Elbow height	Perpendicular distance between the olecranon process and the ground during the preparatory phase of the shooting motion divided by the participant’s height.
Release height	Perpendicular distance between the hand and the ground at the time point of the ball release divided by the participant’s height.
Trunk lean	Angle between the torso in relation to an imaginary vertical axis at the time point of the ball release. A positive value indicates forward lean and a negative value indicates backward lean.
Forearm angle	Angle between the forearm and an imaginary vertical axis. A positive value represents the amount of lateral elbow deviation.

### 2.4. Statistical analysis

Descriptive statistics, mean (standard deviation) or median (interquartile range) were calculated for each biomechanical parameter examined in the present study. Shapiro-Wilk's test and Q-Q plots were used to assess if the assumption of normality was violated. Independent *t*-tests and Mann-Whitney *U* tests were used to examine statistically significant differences between proficient (*n* = 19) and non-proficient free-throw shooters (*n* = 15) as well as differences in biomechanical parameters between made and missed shots within the proficient group of shooters. Participants who made ≥70% of free-throw shots were classified as proficient and the ones that made <70% were classified as non-proficient (14, 15). Cohen's *d* was used to calculate the effect size magnitude: 0.2—small effect, 0.5—moderate effect, and >0.8—large effect (29). Statistical significance was set *a priori* to *p* < 0.05. All statistical analyses were completed with SPSS (Version 27.0; IBM Corp., Armonk, NY, USA).

## 3. Results

Descriptive statistics for each dependent variable examined in the present study are reported in Tables 2, 3. Proficient free-throw shooters exhibited lower knee and COM peak and mean velocities (*d* = 0.425–1.037). No statistically significant differences were observed in any other dependent variables of interest during the preparatory and release phases of the free-throw shooting motion, except release height and trunk lean. Proficient shooters attained greater release height (*d* = 0.438) and had less forward trunk lean (*d* = 0.880) at the time point of ball release when compared to non-proficient shooters. See Table 2.

**Table 2:** Descriptive statistics, mean (standard deviation) or median (interquartile range), for differences in biomechanical parameters between proficient (≥70%) and non-proficient (<70%) free-throw shooters.

Variable (unit)	Non-proficient	Proficient	*p*-value	ES
Release angle (deg)^a^	52.1 (5.4)	51.4 (3.2)	0.179	0.235 (S)
Knee angle—PP (deg)	107.3 (14.9)	113.3 (9.1)	0.183	0.486 (M)
Knee angle—RP (deg)	164.5 (7.9)	165.8 (5.4)	0.549	0.192 (S)
Knee ROM (deg)	57.2 (13.6)	52.5 (9.1)	0.240	0.406 (S-M)
Knee peak angular velocity (deg/s)*	269.4 (60.6)	212.9 (47.6)	0.005	1.037 (L)
Knee mean angular velocity (deg/s)^a,^*	170.7 (43.2)	123.0 (65.6)	0.012	0.425 (S-M)
Hip angle—PP (deg)	150.5 (15.0)	152.1 (8.6)	0.710	0.131 (S)
Hip angle—RP (deg)	173.3 (4.2)	173.6 (4.2)	0.869	0.071 (S)
Hip ROM (deg)	22.8 (13.5)	21.4 (9.1)	0.722	0.122 (S)
Hip peak angular velocity (deg/s)	142.1 (64.9)	111.5 (33.8)	0.113	0.591 (M)
Hip mean angular velocity (deg/s)	57.1 (31.6)	44.2 (17.4)	0.171	0.506 (M)
Ankle angle—PP (deg)	54.5 (6.8)	57.3 (6.9)	0.244	0.409 (S-M)
Ankle angle—RP (deg)	100.4 (13.2)	107.1 (10.6)	0.107	0.559 (M)
Ankle ROM (deg)	45.9 (14.3)	49.8 (12.9)	0.405	0.286 (S)
Ankle peak angular velocity (deg/s)	264.1 (98.1)	241.2 (62.9)	0.416	0.278 (S)
Ankle mean angular velocity (deg/s)	129.0 (59.3)	111.8 (44.3)	0.339	0.329 (S)
Elbow angle—PP (deg)^a^	78.0 (27.4)	85.1 (20.6)	0.471	0.128 (S)
Elbow angle—RP (deg)^a^	159.8 (1.3)	159.6 (1.4)	0.336	0.167 (S)
Elbow ROM (deg)^a^	82.8 (29.4)	73.6 (20.9)	0.430	0.140 (S)
Elbow peak angular velocity (deg/s)	975.2 (131.9)	899.0 (110.1)	0.075	0.627 (M)
Elbow mean angular velocity (deg/s)	232.4 (107.7)	183.7 (86.1)	0.152	0.499 (M)
COM height—PP (m)	0.52 (0.04)	0.53 (0.03)	0.214	0.441 (M)
COM height—RP (m)^a^	0.67 (0.02)	0.67 (0.01)	0.242	0.207 (S)
COM change (m)	0.14 (0.04)	0.14 (0.03)	0.577	0.174 (S)
COM—peak velocity (m/s)*	1.07 (0.23)	0.87 (0.17)	0.007	0.988 (L)
COM—mean velocity (m/s)*	0.69 (0.24)	0.54 (0.15)	0.036	0.733 (M-L)
Stance width—PP (m)	30.0 (7.2)	32.4 (4.9)	0.244	0.390 (S)
Stance alignment—PP (m)	8.1 (6.3)	7.6 (7.0)	0.853	0.075 (S)
Elbow height—PP (deg)^a^	0.57 (0.08)	0.61 (0.12)	0.147	0.253 (S)
Release height (deg)^a,^*	1.12 (0.07)	1.17 (0.05)	0.010	0.438 (S-M)
Trunk lean (deg)*	1.87 (3.28)	−1.11 (3.49)	0.016	0.880 (L)
Forearm angle—PP (deg)	35.2 (6.9)	35.3 (8.3)	0.971	0.013 (S)
Forearm angle—RP (deg)	13.1 (4.8)	13.6 (4.7)	0.726	0.105 (S)
Forearm ROM (deg)^a^	22.5 (16.4)	18.6 (10.2)	0.681	0.074 (S)

ES, effect size (magnitude interpretation); S, small effect; M, medium effect; S-M, small to moderate; L, large effect; M-L, moderate to large; PP, preparatory phase of the shooting motion; RP, release phase of the shooting motion; COM, center of mass; ROM, range of motion.

^a^ Non-normally distributed variables.

* Statistically significant difference between proficient and non-proficient shooters (*p* < 0.05).

**Table 3:** Descriptive statistics, mean (standard deviation) or median (interquartile range), for differences in biomechanical parameters between made and missed free-throw shots within the proficient (≥70%) group of shooters.

Variable (unit)	Missed	Made	*p*-value	ES
Release angle (deg)	52.0 (6.0)	51.5 (4.9)	0.644	0.091 (S)
Knee angle—PP^a^	114.8 (15.1)	118.0 (12.7)	0.286	0.081 (S)
Knee angle—RP (deg)^a^	167.6 (4.4)	167.7 (4.4)	0.856	0.014 (S)
Knee ROM (deg)^a^	51.4 (10.9)	49.5 (10.5)	0.248	0.088 (S)
Knee peak angular velocity (deg/s)^a^	234.1 (78.3)	220.2 (67.8)	0.121	0.118 (S)
Knee mean angular velocity (deg/s)^a^	140.3 (27.9)	138.7 (38.2)	0.209	0.095 (S)
Hip angle—PP (deg)^a^	148.4 (14.6)	149.2 (13.9)	0.778	0.021 (S)
Hip angle—RP (deg)^a^	177.5 (7.3)	177.5 (6.4)	0.791	0.020 (S)
Hip ROM (deg)^a^	26.0 (14.6)	26.1 (13.4)	0.648	0.035 (S)
Hip peak angular velocity (deg/s)^a^	180.4 (127.1)	158.8 (87.9)	0.231	0.091 (S)
Hip mean angular velocity (deg/s)^a^	68.9 (51.6)	66.5 (36.8)	0.784	0.021 (S)
Ankle angle—PP (deg)	57.9 (6.8)	58.5 (5.9)	0.608	0.094 (S)
Ankle angle—RP (deg)^a^	111.2 (12.0)	109.8 (12.0)	0.880	0.011 (S)
Ankle ROM (deg)	50.7 (14.6)	50.6 (12.1)	0.998	0.007 (S)
Ankle peak angular velocity (deg/s)	248.3 (64.9)	247.1 (59.8)	0.919	0.019 (S)
Ankle mean angular velocity (deg/s)	137.7 (33.5)	136.6 (30.2)	0.851	0.034 (S)
Elbow angle—PP (deg)^a^	78.5 (21.4)	81.5 (17.8)	0.621	0.038 (S)
Elbow angle—RP (deg)^a^	168.3 (11.3)	166.1 (12.9)	0.359	0.069 (S)
Elbow ROM (deg)	88.2 (15.6)	85.0 (15.3)	0.288	0.207 (S)
Elbow peak angular velocity (deg/s)^a^	791.5 (113.1)	772.8 (129.4)	0.951	0.005 (S)
Elbow mean angular velocity (deg/s)	248.1 (52.0)	236.5 (52.4)	0.253	0.222 (S)
COM height—PP (m)^a^	0.55 (0.04)	0.55 (0.03)	0.474	0.055 (S)
COM height—RP (m)^a,^*	0.68 (0.02)	0.67 (0.02)	0.021	0.176 (S)
COM change (m)^a^	0.13 (0.04)	0.12 (0.04)	0.414	0.062 (S)
COM—peak velocity (m/s)^a^	0.93 (0.23)	0.90 (0.24)	0.273	0.083 (S)
COM—mean velocity (m/s)	0.64 (0.11)	0.61 (0.11)	0.209	0.249 (S)
Stance width—PP (m)^a^	32.7 (7.5)	33.4 (7.4)	0.392	0.065 (S)
Stance alignment—PP (m)^a^	5.6 (5.4)	5.5 (9.3)	0.824	0.017 (S)
Elbow height—PP (deg)^a^	0.69 (0.18)	0.70 (0.13)	0.957	0.004 (S)
Release height (deg)^a,^*	1.19 (0.04)	1.17 (0.05)	0.035	0.161 (S)
Trunk lean (deg)	−1.58 (3.4)	−1.32 (3.0)	0.674	0.081 (S)
Forearm angle—PP (deg)^a^	33.7 (11.2)	31.2 (9.4)	0.682	0.031 (S)
Forearm angle—RP (deg)	9.6 (4.7)	9.7 (4.8)	0.944	0.021 (S)
Forearm ROM (deg)^a^	22.0 (9.9)	21.1 (8.4)	0.650	0.035 (S)

ES, effect size (magnitude interpretation); S, small effect; PP, preparatory phase of the shooting motion; RP, release phase of the shooting motion; COM, center of mass; ROM, range of motion.

^a^ Non-normally distributed variables;

* Statistically significant difference between proficient and non-proficient shooters (*p* < 0.05).

When examining differences between made and missed shots within the proficient group of free-throw shooters, no statistically significant differences were observed for any dependent variables of interest, except for the release height (*d* = 0.161) and COM height (*d* = 0.176) at the time point of ball release. Despite being small in magnitude, both biomechanical parameters demonstrated greater values for made when compared to missed free-throw shots. See Table 3.

## 4. Discussion

When examining differences in biomechanical characteristics based on free-throw shooting proficiency, the findings of the present study reveal that proficient shooters demonstrated significantly lower knee and COM peak and mean velocities than non-proficient shooters. Also, a notable difference between these two groups of shooters was observed at the timepoint of the ball release (i.e., release phase), where proficient free-throw shooters attained greater release height and had less forward trunk lean when compared to non-proficient shooters. On the other hand, when examining differences based on the outcome of the shooting motion within a group of proficient shooters, our results indicate that made free-throw shots were characterized by lower release and COM heights, while no significant differences were observed in any other variables of interest examined in the present investigation. To the best of our knowledge, this is the first study that implemented an innovative markerless motion capture technology to obtain a deeper insight into a broad spectrum of biomechanical parameters that depict successful free-throw shooting performance.

Previous research has found that higher values of total movement in the knee joint, attained by less knee flexion during the preparatory phase and greater knee extension during the release phase of the free-throw shooting motion, was positively associated with a number of made baskets during the learning process in college-age novice basketball players (10). These findings are contradictory to the results of the present investigation where no differences in knee ROM and angle during preparatory and release phases of the shooting motion were observed between proficient and non-proficient shooters. This discrepancy may be mainly attributed to the participant's skill level, as our study examined a cohort of basketball players with a considerable amount of playing experience (e.g., high school, collegiate). Further, it has been found that proficient free-throw shooters tend to attain greater knee, hip, ankle, and elbow flexion during the preparatory phase of the shooting motion, as well as lower elbow positioning (12, 13). Interestingly, the findings of the present study revealed no significant differences between proficient and non-proficient shooters in any of the aforementioned biomechanical parameters. However, rather than diminishing their impact on the success of the free-throw shooting outcome, we may assume that non-proficient shooters in the present study already incorporated these adjustments in shooting form. Also, based on the shooting proficiency level, it should be noted that the observed magnitudes of the differences in knee, hip, ankle, and elbow flexion were small to moderate (*d* = 0.131–0.486). While not examined in the previously mentioned research reports (12, 13), a similar notion can be made for the same variables captured at the time point of the ball release (*d* = 0.071–0.559). Thus, the observed inconsistencies suggest that there are other performance metrics that may be more sensitive in detecting differences in free-throw shooting proficiency (e.g., joint velocities).

Based on the findings of the present study, some of the key biomechanical parameters capable of differentiating proficient from non-proficient free-throw shooters were knee peak and mean velocities. Proficient shooters had significantly lower knee peak and mean velocities than non-proficient shooters, with differences being moderate to large in magnitude (*d* = 0.425–1.037). Also, COM mean and peak angular velocities were notably lower within the proficient group of shooters (*d* = 0.733–0.988). Yet, considering that no other statistically significant joint velocity-related changes were detected between the two groups of shooters (e.g., hip mean angular velocity, ankle peak angular velocity), we can assume that the decrease in COM peak and mean velocities was primarily driven by a decrease in knee peak and mean velocities. On the other hand, while further research is warranted to examine the optimal velocity ranges, Hudson (30) has indicated that the free-throw shot is a complex basketball-specific motion that requires sub-maximal velocities for most populations of players. In the same investigation the author implied that due to sub-maximal velocity demands, there is an endless number of body segmental contributions that could lead to the ball passing through the basket (30). Our results seem to offer further support to the aforementioned findings, as lower peak and mean velocities showed to be a characteristic of proficient free-throw shooters. Despite not reaching the level of statistical significance, it should be noted that hip, ankle, and elbow peak and mean velocities observed in this study were lower within the proficient than non-proficient group of free-throw shooters (*d* = 0.278–0.627). Overall, our results imply that proficient shooters tended to execute the free-throw shooting motion in a more controlled manner without overemphasizing upper and/or lower-body joint velocities.

Another interesting observation based on the findings of the present study pertains to proficient free-throw shooters being able to attain greater release height and less forward trunk lean at the time point of the ball release when compared to non-proficient shooters (*d* = 0.438–0.880). Previous research has documented the importance of the higher point of the ball release for a successful outcome of the free-throw shooting motion (15, 16). As the release height increases, the launching speed becomes smaller, which ultimately allows players to shoot with a greater margin of error (15). Further, despite addressing teaching points for optimizing jump shots performance, Knudson (28) has suggested that skilled shooters are capable of minimizing horizontal body movement and optimizing near-vertical trunk alignment. So, we can conclude that the aforementioned findings directly align with the results obtained in the present investigation as well. In addition, it should be noted that an increase in release height is often attributed to an increase in release angle and/or vertical jump displacement (27, 31). Interestingly, that was not the case in the present study, as both release angle and COM height were similar between proficient and non-proficient free-throw shooters. Moreover, the release angle for both groups of shooters was identical to previously determined optimal release conditions (21). Thus, we can assume that the observed increase in the release height can be largely attributed to having less forward trunk lean (i.e., keeping the torso in a near vertical position) at the time point of the ball release.

Using an innovative markerless motion capture system allowed for the analysis of the foot placement (i.e., stance width and alignment) as well as the forearm angle during both preparatory and release phases of the free-throw shooting motion. Our results revealed no statistically significant differences between the two groups based on the level of shooting proficiency (*d* = 0.075–0.390). Both proficient and non-proficient shooters tended to place the right foot slightly in front of the left during the preparatory phase of the free-throw shooting motion while keeping similar stance width (i.e., 30.0–32.4 cm). On the other hand, the forearm ROM and angle during both preparatory and release phases of the free-throw shooting motion were almost identical based on a level of shooting proficiency. Although smaller in magnitude, previous research has found that proficient shooters had less lateral elbow deviation during the preparatory phase of the shooting motion (15). As previously mentioned, this discrepancy may be attributed to the differences in the skill level of participants, as well as individual differences in shooting form (30).

When examining differences between made and missed shots within the proficient group of free-throw shooters, the only variables that reached the level of statistical significance from a large pool of biomechanical parameters examined in the present study were release height and COM height at the time point of the ball release. Surprisingly, both variables were greater for missed than made shots (*d* = 0.161–0.176). These findings seem to contradict the previously discussed benefits of greater release height for the improvement in free-throw shooting accuracy (15). However, at the same time, they indicate that overemphasizing release height and vertical jump displacement may be counterproductive for eliciting improvements in free-throw shooting accuracy. Similar observations emerged from a recently published study where no differences between made and missed free-throw shots were found in elbow, hip, knee, and ankle angles, as well as the elbow height during the preparatory phase of the shooting motion (12). Moreover, despite being focused on analyzing kinetic and kinematic characteristics of mid-range jump shots in college-age male basketball players, the same group of authors found no difference in vertical jump displacement, entry angle, maximal trajectory height, impulse, and peak concentric and landing forces between made and missed mid-range two-point shooting attempts (32). Considering that the majority of differences in biomechanical parameters of interest examined in the present study between made and missed free-throw shots are trivial to small in magnitude (*d* = 0.005–0.222), it is obvious that there are other factors that have a substantial impact on shooting accuracy. Some of them might be wrist mechanics and kinematic chaining that have not been extensively studied in the scientific literature and warrant further investigation.

While allowing sports scientists and coaches to obtain a deeper insight into the biomechanical characteristics of free-throw shooting motion, this study is not without limitations. The testing procedures were performed in a laboratory-based environment with no audience and were non-fatiguing in nature, which does not directly resemble in-game basketball requirements. Also, future research should focus on examining if these findings are gender-specific and if they remain applicable to other levels of basketball competition (e.g., amateur, professional).

## 5. Conclusion

In conclusion, the findings of the present study reveal that proficient free-throw shooters performed the shooting motion in a more controlled manner by having significantly lower knee and COM peak and mean velocities. Also, proficient shooters attained a significantly greater release height and had less forward trunk lean when compared to non-proficient shooters at the time point of the ball release. Moreover, despite being beneficial for improvements in shooting accuracy, our findings suggest that overemphasizing the release height may be in certain instances counterproductive, as it may lead to more missed than made free-throw shots within the proficient group of shooters.

## Data Availability

The raw data supporting the conclusions of this article will be made available by the authors, without undue reservation.
